# Epigenetic Regulation of Heat Stress in Plant Male Reproduction

**DOI:** 10.3389/fpls.2022.826473

**Published:** 2022-02-10

**Authors:** Shikha Malik, Dazhong Zhao

**Affiliations:** Department of Biological Sciences, University of Wisconsin-Milwaukee, Milwaukee, WI, United States

**Keywords:** heat stress, male reproduction, anther, tapetum, and pollen development, epigenetic regulation, DNA methylation

## Abstract

In flowering plants, male reproductive development is highly susceptible to heat stress. In this mini-review, we summarized different anomalies in tapetum, microspores, and pollen grains during anther development under heat stress. We then discussed how epigenetic control, particularly DNA methylation, is employed to cope with heat stress in male reproduction. Further understanding of epigenetic mechanisms by which plants manage heat stress during male reproduction will provide new genetic engineering and molecular breeding tools for generating heat-resistant crops.

## Introduction

Short- and long-term heat stress have detrimental effects on overall growth and development in plants (Kotak et al., 2007); however, reproductive organs, particularly the male reproductive organ, are more susceptible to elevated temperatures comparing with vegetative organs (Abiko et al., 2005; Sakata et al., 2010; Sato et al., 2014, 2019; Fragkostefanakis et al., 2016; Begcy et al., 2019; He et al., 2019). Heat stress leads to partial or complete male sterility, which in turn causes yield loss in crops (Smith and Zhao, 2016). Being sessile, plants employ various mechanisms to cope with heat stress. Besides the genetic control, transcriptome and genome-wide DNA methylation analyses have revealed that the epigenetic regulation plays a pivotal role in reprogramming expression of genes required for plants to manage heat stress during reproductive development. In this mini-review, we focus on discussing research in epigenetic mechanisms underlying heat stress response in male reproduction.

## Plant Male Reproduction Is Highly Sensitive to Heat Stress

**Figure 1:**
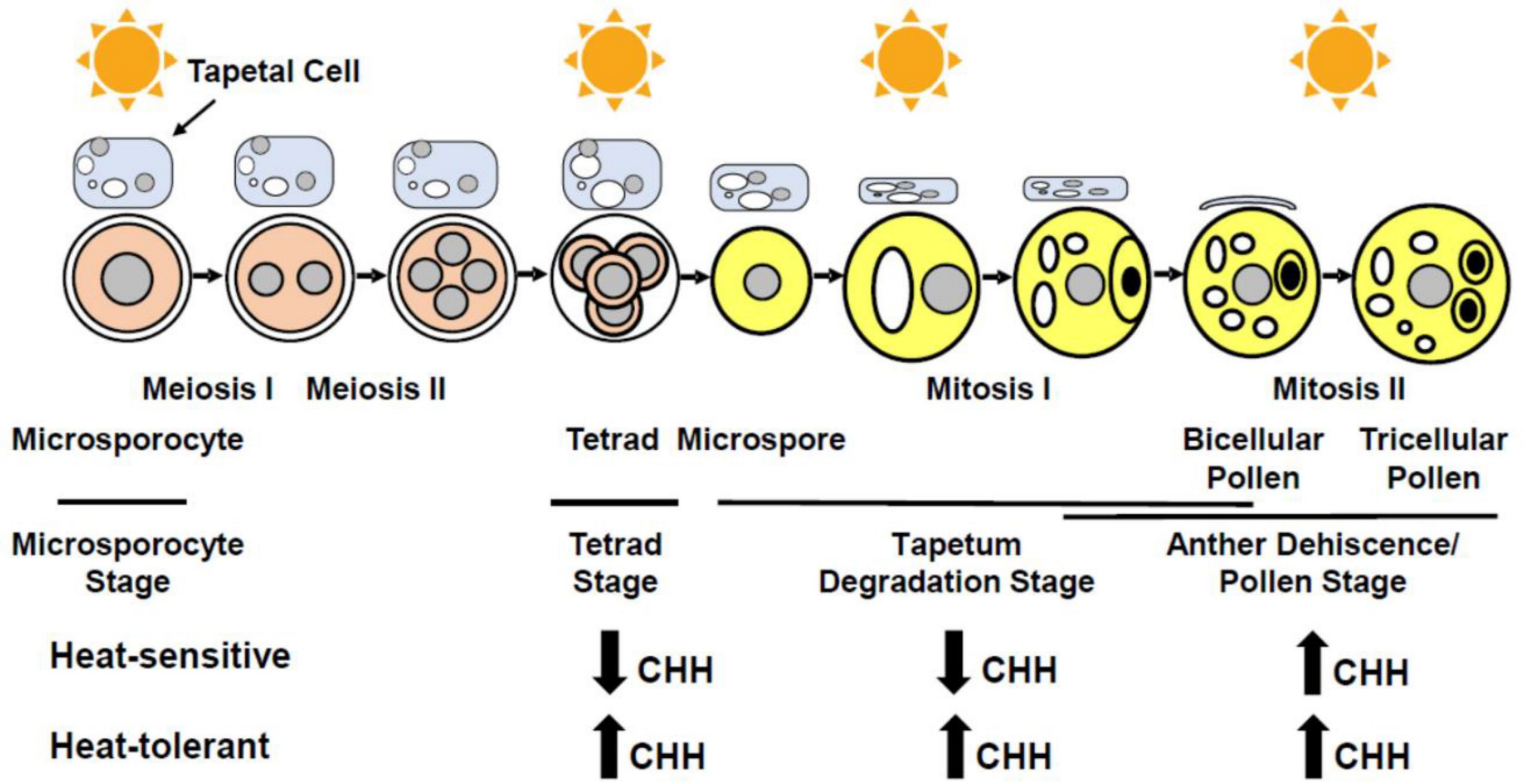
Schematic representation of stages susceptible to heat stress (indicated by sun symbols) during male reproduction and their methylation patterns. CHH methylation is decreased under heat stress at tetrad and tapetum degradation stages in anthers of heat-sensitive plants. CHH methylation is increased under heat stress at tetrad and tapetum degradation stages in anthers of heat-tolerant plants, as well as at the anther dehiscence/pollen stage in anthers of both heat-sensitive and -tolerant plants (H in CHH representing A, T, or G).

Heat stress impairs anther wall cell differentiation, microsporogenesis, and pollen formation, resulting in partial or complete male sterility in various plants. Stamen is the male reproductive organ of flowering plants, comprising of an anther where pollen (the male gametophyte) develops and a filament that anchors the anther to the flower. A typical anther has four lobes (microsporangia; Goldberg et al., 1993; Zhao, 2009; Feng et al., 2013; Walbot and Egger, 2016); within each lobe, the central pollen mother cells (PMC or microsporocytes) are surrounded by four concentrically organized layers of somatic cells: the epidermis, endothecium, middle layer, and tapetum (outside to inside). PMCs give rise to pollen *via* a series of events. PMCs undergo meiosis to produce tetrads that release microspores. After two rounds of mitosis, microspores eventually become pollen grains which contain a vegetative cell and two sperm cells (Sanders et al., 1999; Figure 1). The somatic anther wall cells, particularly tapetal cells (tapetum), are essential for the normal development and release of pollen. Tapetum, consisting of a monolayer or multilayers of endopolyploid cells, which is associated with successive stages of PMC, tetrads, microspores, and developing pollen as anther development progresses (Goldberg et al., 1993; Scott et al., 2004; Walbot and Egger, 2016; Figure 1). Early on, tapetal cells secrete enzymes required for releasing haploid microspores from tetrads (Pacini et al., 1985; Clément and Pacini, 2001; Hsieh and Huang, 2007; Ishiguro et al., 2010; Parish and Li, 2010). Later, tapetal cells provide energy and materials for pollen development and pollen coat formation (Wu et al., 1997; Wang et al., 2003; Parish and Li, 2010; Huang et al., 2017). Lack of a tapetum or an abnormal tapetum impairs microspore and pollen development, causing male sterility (Mariani et al., 1990; Zhao et al., 2002; Zhang et al., 2014). Furthermore, endothecium is necessary for anther dehiscence (Cecchetti et al., 2013; Murphy et al., 2015).

Heat stress causes male sterility and seed yield loss are mainly ascribed to aberrant tapetum and pollen development (Parish et al., 2012; De Storme and Geelen, 2014). Decreased pollen viability due to heat stress has been reported in many crops, such as common bean (Gross and Kigel, 1994; Prasad et al., 2002), rice (Endo et al., 2009), cotton (Min et al., 2014; Song et al., 2015), tomato (Pressman et al., 2002; Giorno et al., 2013), pepper (Erickson and Markhart, 2002), wheat (Saini and Aspinall, 1982; Saini et al., 1984), barley (Sakata et al., 2010), cowpea (Ahmed et al., 1992), peanut (Vara Prasad et al., 1999; Zoong Lwe et al., 2020), and flax (Cross et al., 2003; Table 1). In crops, such as wheat, episodes of male sterility were observed upon 3 days of treatment at 30/30°C (day/night, the same thereafter) during meiosis, and irregular tapetum degeneration is a plausible cause for pollen abortion (Saini et al., 1984). In heat-sensitive wheat varieties, elevated temperature (35/24°C) caused tapetum degradation and pollen abortion (Browne et al., 2021). Premature pollen development in common bean at 33/29°C is also a result of early tapetum degeneration (Suzuki et al., 2001). Furthermore, abnormally wavy, looped endoplasmic reticulum (ER) structures were detected in heat-stressed tapetal cells (Suzuki et al., 2001), suggesting that ER malfunction in tapetal cells might cause male sterility under heat stress (De Storme and Geelen, 2014). Heat stress results in DNA fragmentation, cytoplasmic shrinkage, and vacuolation in early tapetal cells of thermosensitive genic male-sterile (TGMS) rice, suggesting that the precocious programmed cell death (PCD) of tapetal cells during heat stress causes male sterility (Ku et al., 2003). Impaired tapetal cells by heat stress also affects callose degradation in PMCs and pollen wall formation, such as exine patterning (Suzuki et al., 2001; Parish et al., 2012; Djanaguiraman et al., 2014). Moderately high temperature (30/25°C) causes aberrant mitochondria, ER, and nuclear membranes in PMCs (Oshino et al., 2007). Moreover, abnormal meiosis occurred in PMCs in heat-stressed wheat (Omidi et al., 2014). Recently, abnormal cross-over was observed in *Arabidopsis* male meiocytes under high temperature (De Storme and Geelen, 2020). Heat stress (36–38°C) also impaired chromosome segregation and cytokinesis during male meiosis in *Arabidopsis* (Lei et al., 2020). Moreover, acute heat stress on *Arabidopsis* causes defects in male germline and sporophytic anther tissues (Hedhly et al., 2020). A recent report showed that pollen abortion was subjected to heat stress (35/25°C) at the pre-meiotic stage in maize with downregulated *MAGO* (*MALE*-*ASSOCIATED ARGONAUTE*-*1* and -*2*) genes (Lee et al., 2021). Further studies revealed that heat stress induced MAGO hypophosphorylation which affects accumulation of 21-nt phasiRNAs and then the activity of retrotransposons in anther wall cells. Thus, the surveillance mechanism mediated by Argonaute is important for protecting male sterility under heat stress.

**Table 1:** Effects of heat stress on plant male reproduction.

Plant	Temperature	Effect	Reference
*Arabidopsis* (*Arabidopsis thaliana*)	30–32°C 6 to 48 h; 36–38°C; 24 h	Abnormal anther wall, male meiosis, male germline, and meiotic cytokinesis	De Storme and Geelen, 2020; Lei et al., 2020
Cotton (*Gossypium hirsutum*)	35–39°C 7 days and 40/34°C	Abnormal microspores, tapetum, and pollen grains	Min et al., 2014; Song et al., 2015; Ma et al., 2018
Barley (*Hordeum vulgare*)	30/25°C (day/night) 5 days	Abnormal pollen mother cell and tapetum	Abiko et al., 2005; Oshino et al., 2007; Sakata et al., 2010
Rice (*Oryza sativa*)	39/30°C (day/night) and 32°C	Decreased pollen viability, premature tapetum degradation in TGMS rice	Ku et al., 2003; Endo et al., 2009; Zhao et al., 2018
Maize (*Zea mays*)	35/25°C (day/night) 3 days	Decreased pollen viability	Lee et al., 2021
Tomato (*Lycopersicon esculentum*)	36/26°C (day/night) 3 days and 32/26°C	Aberrant male gametogenesis, decreased pollen grain viability	Pressman et al., 2002; Giorno et al., 2013
Wheat (*Triticum aestivum*)	>30 and 30°C for 3 days	Abnormal anthers, tapetum degradation, sporogenesis, and pollen grain viability	Saini and Aspinall, 1982; Saini et al., 1984; Omidi et al., 2014; Browne et al., 2021
Bean (*Phaseolus vulgaris*)	32.7 and 32/27°C (1 or 5 days)	Abnormal pollen grains and tapetum	Gross and Kigel, 1994; Suzuki et al., 2001
Cowpea (*Vigna unguiculata*)	33/20°C or 33/30°C (day/night)	Tapetum, tetrads disorganized, Abnormal pollen grains	Ahmed et al., 1992
Bell pepper (*Capsicum annuum*)	36°C	Deformed pollen grains	Erickson and Markhart, 2002
*Brachypodium distachyon*	36°C	Aborted uninucleate, vacuolated microspore, ruptured tapetal cells, Abnormal pollen grains	Harsant et al., 2013
Flax (*Linum usistatissimum*)	Increase of 3°C per hour to 40°C for 7 h, held for 2 h at 40°C	Compressed and folded pollen grains	Cross et al., 2003
Grain sorghum (*Sorghum bicolor*)	36/26°C and 38/28°C for 10 days	Reduced pollen germination	Jain et al., 2007; Djanaguiraman et al., 2014
Peanut (*Arachis hypogaea*)	28, 34, 42, and 48°C	Pollen viability	Vara Prasad et al., 1999; Zoong Lwe et al., 2020

Anther wall cells and pollen in tomato plants upon heat stress (32/26°C) witness decreased starch and soluble sugar contents (Pressman et al., 2002). In sorghum, heat-stressed (36/26°C) microspores also showed reduced starch content and sucrose deficiency, thus reducing pollen germination (Jain et al., 2007). Moreover, an imbalance in ROS (reactive oxygen species) homeostasis in tapetal cells due to heat stress possibly causes early PCD of tapetal cells (De Storme and Geelen, 2014). In rice anthers, ROS and superoxide dismutase (SOD) are significantly increased at the male meiosis stage (Zhao et al., 2018). In barley, male sterility is possibly attributed to the hyper-phosphorylation of the serine-5 residue at the C-terminal domain of RNA Polymerase (RNA Pol) II, which alters expression of many genes during early anther development under high-temperature conditions (Abiko et al., 2005). Furthermore, auxin synthesis in *Arabidopsis* and barley anthers are reduced during high temperatures, whereas exogenous application of auxin to anthers improved pollen thermotolerance in barley (Sakata et al., 2010; Higashitani, 2013). Auxin biosynthesis genes, such as *YUCCA*-*YUC2* and *YUC6*, were suppressed in anthers exposed to high temperatures (33°C; Sakata et al., 2010). Heat stress generally alters expression of various genes which affect cell proliferation, photosynthesis, hormones, starch metabolism, heat shock response, and ROS production (Yang et al., 2006; Yamakawa et al., 2007; Endo et al., 2009; Frank et al., 2009; Bita et al., 2011; Mangelsen et al., 2011; Guan et al., 2013; Min et al., 2014; Song et al., 2014; Fragkostefanakis et al., 2015; González-Schain et al., 2016; Zhang et al., 2017; Zhao et al., 2018; Begcy et al., 2019; Qian et al., 2019b). Here we mainly discuss the epigenetic mechanisms by which plants respond to heat stress during male reproduction.

## Epigenetic Modifications During Heat Stress Response

In contrast with the molecular mechanisms underlying heat stress at the transcriptional level, epigenetic regulation during high-temperature stress is not well understood in plants (Ohama et al., 2017). Different plant organs/cells have been studied to understand the role of epigenetic modifications during heat stress. For instance, exposure of soybean root hairs and roots stripped root hairs to heat stress (40°C) caused hypomethylation of CHH (H = A, T or C; Hossain et al., 2017). Heat stress also induced hypomethylation of CG and CHG in cultured microspores of *Brassica napus* (Li et al., 2016). In maize seedlings, 325 differentially methylated genes (DMG) were identified responding to heat stress (42°C). Interestingly, 9 DMG associated with spliceosome showed the decreased methylation level during heat stress (Qian et al., 2019a). Moreover, the *Brassica napus* heat-sensitive genotype possesses a higher level of DNA methylation than the heat-tolerant genotype during heat stress (37–45°C; Gao et al., 2014). Collectively, these findings reveal that DNA methylation is responsive to heat stress. The effect of heat stress on methylation in various plants is summarized in Table 2.

**Table 2:** Methylation patterns in plants during heat stress.

Plant	Temperature	Tissue	Methylation pattern	Reference
Soybean (*Glycine max*)	40°C	Roots	Hypomethylation CHH context	Hossain et al., 2017
Rapeseed (*Brassica napus*)	37°C for 2 h and 45°C for 3 h	Seedling	Hypermethylation in heat-sensitive variety	Gao et al., 2014
Maize (*Zea mays*)	42°C for 8 h	Seedlings	Reduced methylation of 9 differentially methylated genes	Qian et al., 2019a
Rapeseed (*Brassica napus* cv. Topas)	32°C for 6 h	Cultured Microspores	Hypomethylation CG and CHG context	Li et al., 2016
Arabidopsis (*Arabidopsis thaliana*)	42°C	Leaves	Decreased DNA methylation	Korotko et al., 2021
Cotton (*Gossypium hirsutum*)	35°C to 39/29°C to 31°C day/night for 7 days	Anthers	Hypomethylation in heat-sensitive variety	Min et al., 2014; Ma et al., 2018

Genes involved in DNA methylation, histone modification, chromatin modeling, and small RNA biogenesis were studied for their roles in response to heat stress. Loss-of-function mutant of the *NUCLEAR RNA POLYMERASE D2A* (*NRPD2*) gene which encodes the second largest subunit of RNA POL IV and POL V is sensitive to heat stress (Popova et al., 2013). A RPD3-type of histone deacetylase mutant *hda6* is sensitive to heat stress. In contrast, DNA methyltransferase mutants, such as *domains rearranged methylase1* (*drm1*), *domains rearranged methylase2* (*drm2*), and *chromomethylase3*(*cmt3*), presented less pronounced response to heat stress (Popova et al., 2013). Interestingly in wild-type *Arabidopsis* plants, heat stress induced expression of the key DNA methyltransferase gene *DRM2* as well as *NUCLEAR RNA POLYMERASE D1A* (*NRPD1*) and *NUCLEAR RNA POLYMERASE D1B* (*NRPE1*) which encode the largest subunit of RNA Pol IV and RNA Pol V, respectively (Naydenov et al., 2015). Conversely, the prolonged heat exposure decreased expression of DNA methyltransferase genes *METHYLASE1* (*MET1*) and *CHROMOMETHYLASE3*(*CMT3*; Naydenov et al., 2015). The *DRM2* expression during heat stress might be regulated by RNA Pol IV and/or RNA Pol V (Naydenov et al., 2015).

DNA methylation associated with NRPD2 and histone modification mediated by HDA6 might play different roles in transcriptional reprogramming for coping with heat stress. Transcriptomic analysis of directly heat-stressed *hda6* mutants revealed a larger set of mis-regulated genes comparing with the heat-stressed *nrpd2* mutant, while after recovery from heat stress a much broader transcriptional response was detected in *nrpd2* mutants than *hda6* mutants and wild-type plants (Popova et al., 2013). In *hda6* mutants, mis-regulated genes are involved in diverse functions, such as protein processing, hormone signaling, vegetative and reproductive development, transport, and metabolism; however, GO enrichment analysis found that mis-regulated genes in *nrpd2* mutants were associated with starch catabolism, fatty acid oxidation, abiotic stress response, and auxin and cytokinin signaling pathways. A little overlap of mis-regulated gene sets between *hda6* and *nrpd2* mutants suggests that *HDA6* and *NRPD2* function differently at different stages of heat response (Popova et al., 2013). Similarly, in the heat-stressed (42°C) maize seedling, some of the key KEGG pathway enrichment involve spliceosome, RNA transport, ubiquitin-mediated proteolysis, and carbon metabolism (Qian et al., 2019a), suggesting that heat stress affects a diverse range of biological pathways which might be regulated *via* the epigenetic control.

Heat stress activates the *ONSEN* (“hot spring” in Japanese) retrotransposon and synthesis of extrachromosomal DNA copies in *Arabidopsis* seedlings (Ito et al., 2011). Heat stress triggers accumulation of *ONSEN* in mutants lacking RNA Pol IV and RDR2, which are main components in the RdDM pathway. Interestingly, the memory of heat stress (i.e., transgenerational inheritance of *ONSEN* insertion) can only occur in the progeny of mutant plants defective in siRNA biogenesis. Heat stress induced epigenetic memory associated with hypermethylation of H3K4me2 and H3K4me3 can be maintained for several days in *Arabidopsis* somatic cells (Lamke et al., 2016). Moreover, transgenerational epigenetic memory induced by heat stress is transmitted *via* HEAT SHOCK TRANSCRIPTION FACTOR A2 (HSFA2) activated H3K27me3 demethylase in *Arabidopsis* (Liu et al., 2019; Yamaguchi et al., 2021). Thus, histone modification is essential for thermotolerance memory.

## Epigenetic Regulation of Heat Stress During Male Reproduction

Besides genetic regulation, the epigenetic control, particularly DNA methylation, is an important mechanism for plants to manage heat stress during male reproduction. RNA-directed DNA methylation (RdDM) in plants involves various components, such as small interfering RNAs (siRNA) and DNA methyltransferase DRM2 (Law and Jacobsen, 2010). Methylation of DNA occurs at specific sites: symmetric patterns of CpG/CpNpG and asymmetric CpNpN. In plants, methylation of asymmetric cytosine (CpNpG) is regulated by *CHROMOMETHYLASE* (*CMT*; Bartee et al., 2001).

Pollen comprises one vegetative nucleus and two sperm nuclei which maintain more stable methylation patterns than leaves and roots (Hsieh et al., 2016). The vegetative nucleus lacks *DECREASE IN DNA METHYLATION 1* (*DDM1*), leading to reactivation of transposable elements. Reduction of DNA methylation in pollen causes transcriptional reprogramming (Slotkin et al., 2009). Cell-specific DNA methylation studies revealed that CG and CHG methylation were retained in microspores and sperm cells, whereas the CHH methylation was lost (Calarco et al., 2012). Interestingly, DNA methylation is reestablished in the vegetative cell *via* siRNA-mediated RdDM (Calarco et al., 2012). Repetitive elements were found to be active during pollen development (Slotkin et al., 2009), while heat stress can activate repetitive elements in *Arabidopsis* seedlings by epigenetic regulation (Pecinka et al., 2010). Most key genes required for DNA methylation, such as *DRM2*, *NRPD1*, and *NRPE1*, are upregulated during heat stress in *Arabidopsis* (Naydenov et al., 2015), supporting the involvement of DNA methylation in heat stress. New findings suggest that sperm cells have asymmetric mCHG, whereas vegetative nuclei and microspores possess symmetric mCHG (Borges et al., 2021). DNA methylation changes during male reproductive development were recently summarized (Papareddy and Nodine, 2021).

Transcriptome studies on heat-treated cotton anthers identified various genes involved in histone modification and DNA methylation. Under heat stress, the heat-tolerant cotton line produces normal anthers and pollen, while the heat-sensitive line is defective in anther dehiscence and fails to form viable pollen. Heat stress decreased expression of *DNA CYTOSINE-5-METHYLTRANSFERASE* (*DRM1*) and *S-ADENOSYL-l-METHIONINE-DEPENDENT METHYLTRANSFERASE* (*DRM3*) at tetrad and tapetum degradation stages in heat-sensitive cotton anthers, while their expression remains similar in heat-tolerant cotton anthers with an exception of increased expression of *DRM3* at the tetrad stage (Min et al., 2014). Similarly, expression of *NEEDED FOR RDR2-INDEPENDENT DNA METHYLATION* (*NERD*), *NUCLEAR RNA POLYMERASE D1B* (*NRPD1B*), and *S-ADENOSYL-L-HOMOCYSTEINE HYDROLASE1* (*SAHH1*), which are required for normal DNA methylation, is suppressed by heat stress in heat-sensitive cotton anthers (Min et al., 2014). During heat stress, heat-sensitive cotton anthers undergo DNA hypomethylation, while heat-tolerant cotton anthers have a high level of DNA methylation. Furthermore, pollen sterility and defects in anther dehiscence are possibly caused by hypomethylation in the heat-sensitive cotton (Ma et al., 2018). Studies on expression changes of genes associated with DNA methylation in cotton anthers under heat stress provide strong evidence that the epigenetic regulation is required for plants to cope with heat stress.

CHH methylation mediated by RdDM showed more prominent changes comparing to CG and CHG methylation, suggesting that heat stress mainly induces the RdDM activity in anthers. Most of heat-induced CHH methylations were found in promoters and downstream regions of protein-coding genes (Ma et al., 2018). Interestingly, the DNA methylation status varies with anther stages upon heat stress. At tetrad, tapetum degradation, and anther dehiscence/pollen stages, the CHH methylation level in heat-tolerant cotton anthers is increased upon heat stress; however, heat-sensitive cotton anthers depicted hypo-CHH methylation patterns at tetrad and tapetum degradation stages, while an increased CHH methylation level at the anther dehiscence/pollen stage during heat stress (Figure 1). Hence, heat stress may affect RdDM function in an anther stage-specific manner (Ma et al., 2018). Heat stress alters the DNA methylation level, which affects expression of genes involved in sugar metabolism and ROS generation. The abnormal concentration of sugar and ROS therefore impairs anther and pollen development. These discoveries shed light on a novel molecular mechanism by which plants ensure the success of male reproduction under high temperature, thus providing new tools for improving crops to adapt to the challenge of global warming.

Long non-coding RNA (lncRNA) is important for male fertility. In rice, an lncRNA named the long-day-specific male-fertility-associated RNA (LDMAR) is essential for pollen development under the long-day condition (Ding et al., 2012). A single nucleotide mutation in *LDMAR* increased CG methylation in the *LDMAR* promoter region, which decreased the *LDMAR* expression and thus induced PCD in anther cells. The lncRNA expression responds to stresses spatially and temporally in plants (Yu et al., 2019). Among 54 putative heat stress-induced lncRNAs, *TahlnRNA27* and *TalnRNA5* were highly upregulated by heat stress in wheat (Xin et al., 2011). Differentially expressed lncRNAs were also observed during heat stress in *Brassica rapa* (Wang et al., 2019), *Brassica juncea* (Bhatia et al., 2020), and maize (Lv et al., 2019). A recent study in *Arabidopsis* showed that 131 pollen-specific intergenic expressed loci (XLOC), which mostly encode lncRNAs, are heat stress responsive (Rutley et al., 2021). These results suggest that lncRNAs might play an important role in heat stress response during male reproduction *via* epigenetic regulation.

MicroRNAs (miRNAs) are another set of non-coding RNAs which are known to regulate gene expression at the post-transcriptional level (Bartel, 2004; Liu et al., 2010; Chen et al., 2016; Huang et al., 2016). In *Brassica rapa* seedlings, heat stress significantly decreased expression of novel miRNAs *bra*-miR1885b.3 and bra-miR5716 (Yu et al., 2011). In barley, heat stress induced expression of miR160a, 166a, 167h, and 5175a, while expression levels of their target genes, such as *AUXIN RESPONSE TRANSCRIPTION FACTORs* (*ARFs*), were reduced upon heat stress (Kruszka et al., 2014). In *Arabidopsis*, the miR398 expression was rapidly induced by heat stress, while its target genes like *CSD* (encoding the copper/zinc SOD) and *CCS* (encoding a chaperone for CSD) were downregulated by heat stress (Guan et al., 2013). Moreover, heat shock factors HSFA1b and HSFA7b are required for heat stress induced the miR398 expression. Furthermore, the heat stress-induced miR156 plays a crucial role in regulating heat stress memory *via* repressing expression of *SPL* (*SQUAMOSA-PROMOTER BINDING-LIKE*) genes (Stief et al., 2014). These results suggest that miRNAs are generally important for heat stress response in plants.

In both heat-tolerant and heat-sensitive cotton anthers, heat stress repressed the miR156 expression, which consequently increased expression of its target *SPL* genes (Ding et al., 2017). The miR160 expression was suppressed in heat-tolerant cotton but increased in heat-sensitive cotton under heat stress. MiR160 target genes *ARF10* and *ARF17* showed opposite expression pattens to miR160. A recent study identified a plethora of miRNAs which respond to heat stress at a stage-specific manner during cotton anther development (Chen et al., 2020). For instance, expression of miR160, miR167, and miR2949 was elevated at the sporogenous cell proliferation stage under high temperature, while miR156 responded to heat stress at male meiosis and microspore release stages. MiRNAs are also involved in epigenetic regulation *via* controlling DNA methylation and histone modification. MiR165/166 mediates methylation of downstream coding sequences of their target genes *PHABULOSA* and *PHAVOLUTA* in *Arabidopsis* (Bao et al., 2004). MiR156 and its target genes *SPLs* control transition from juvenile to adult phase in *Arabidopsis* (Xu et al., 2018; Manuela and Xu, 2020). *MIR156A* and *MIR156C* loci are major contributors to the formation of mature miR156. The H2A histone variant H2A.Z promotes expression of *MIR156A* and *MIR156C via* increasing the H3K4me3 level in these two loci (Xu et al., 2018). Although lacking direct evidence, it is possible that miRNAs cope with heat stress *via* epigenetic regulation during male reproduction in plants.

## Conclusion and Perspectives

Male reproductive development is highly susceptible to episodes of heat stress. Heat stress leads to impaired tapetum, abnormal microspores, and pollen abortion, which cause male sterility in plants and adversely affect yield due to failure or reduction in fertilization. Different plants respond to heat stress differently, which makes it important to identify key stages susceptible to heat stress during male reproduction. This can help take correct measures to protect plants against heat stress at specific stages during plant male reproduction.

At the molecular level, plants respond to heat stress in multiple ways. Molecular genetics, transcriptomic, and proteomic studies identified a wide array of genes and gene networks associated with heat stress during male reproduction in various crops (Giorno et al., 2013; Zhang et al., 2017; Keller and Simm, 2018; Begcy et al., 2019; Liu et al., 2020; Lohani et al., 2020; Chaturvedi et al., 2021). During male reproduction, heat stress not only affects expression of genes controlling epigenetic modifications, but also ultimately alters DNA methylation status. LncRNA and miRNA also appear important for heat stress response during plant male reproductive development, further suggesting that epigenetic control is a critical means for plants to cope with heat stress.

It is imperative to elucidate functional significance of epigenetic modifications and associated genes in heat stress response during male reproduction in economic plants. Tapetal cells, male meiocytes (microsporocytes), microspores, and pollen are sensitive to high temperature (Figure 1). Tapetal cells are special in terms of their endopolyploidy, formation of unique organelles (i.e., elaioplast, tapetosome, and ubisch body), highly active carbohydrate and lipid metabolism, and PCD. Tapetal cells are required for releasing haploid microspores from tetrads and for supplying energy and materials for pollen development and pollen coat formation. Numerous studies using various plants have found that heat stress affects tapetal cell differentiation and degeneration, which consequently leads to abnormal microspores and pollen abortion. Thus, it is necessary to preform single-cell transcriptomic and proteomic analyses to identify genes, gene networks, as well as particularly DNA methylation and histone modification marks that are responsible for heat stress in tapetal cells, male meiocytes, microspores, and pollen. In addition, it would be worthwhile to investigate transgenerational epigenetic effects (epigenetic memory) on heat tolerance during male reproduction in plants. CRISPR-based targeted modification of epigenetic marks has emerged as a powerful tool for improving plant traits, such as heat tolerance (Ghoshal et al., 2021). Although emerging evidence suggests the importance of epigenetic regulation for heat stress response especially during male reproduction, applying the related findings to generating thermotolerant crops *via* genetic engineering and molecular breeding is still a challenge.

## Author Contributions

SM and DZ conceived the idea and wrote the manuscript. All authors contributed to the article and approved the submitted version.

## Funding

The research in the DZ lab is supported by the National Science Foundation (NSF IOS-1322796) and USDA National Institute of Food and Agriculture (NIFA, 2022-67013-36294).

## Conflict of Interest

The authors declare that the research was conducted in the absence of any commercial or financial relationships that could be construed as a potential conflict of interest.

## Publisher’s Note

All claims expressed in this article are solely those of the authors and do not necessarily represent those of their affiliated organizations, or those of the publisher, the editors and the reviewers. Any product that may be evaluated in this article, or claim that may be made by its manufacturer, is not guaranteed or endorsed by the publisher.
